# Experimental study of crack behavior in pressurized high-density polyethylene water pipes

**DOI:** 10.1016/j.mex.2019.04.009

**Published:** 2019-04-16

**Authors:** Syed Ali Sadr-Al-Sadati, Mohammadreza Jalili Ghazizadeh

**Affiliations:** a Faculty of Civil, Water and Environmental Engineering A.C., Shahid Beheshti University, Tehran, Iran; b Faculty of Civil, Water and Environmental Engineering A.C., Shahid Beheshti University, Tehran, Iran, East Vafadar Blvd., Tehranpars, Tehran, P.O. Box: 16765-1719, 1658953571, Iran

**Keywords:** In the present study, longitudinal crack behavior was investigated experimentally by a semi-industrial pilot-scale in HDPE pipes, Leakage-pressure relation, High-density polyethylene (HDPE) pipe, Longitudinal slit, Temperature, Elastic and plastic behavior

## Abstract

Leakage of High-Density Polyethylene pipes are more sensitive to pressure. Longitudinal slits are the most failure in High-Density Polyethylene pipes that occur in water distribution networks. In the present study, longitudinal crack behavior in High-Density Polyethylene pipes is studied by a semi-industrial pilot-scale. Considering that longitudinal slits leakage behavior is a function of variations in leak area that can have elastic or plastic behavior, a new criteria has been introduced for the leak ares variation. The obtained results show that the slits can have plastic behavior even in normal pressures. Also, it is observed that the leakage exponent in the elastic phase is between 0.44 and 1.44 and it increases up to 2 in the plastic phase. Results of this study can be used to estimate the amount of leakage in High-Density Polyethylene pipes and to choose the appropriate leakage reduction strategies.

• A new method is presented for evaluating the effective parameters on leakage in longitudinal slits in High-Density Polyethylene pipes including the water temperature.
• A new method is presented for estimating discharge coefficient and leakage flow in longitudinal slits in High-Density Polyethylene pipes.
• A new method has been introduced to distinguish the elastic or plastic behavior of the longitudinal slits.

## Graphical abstract

**Figure:**
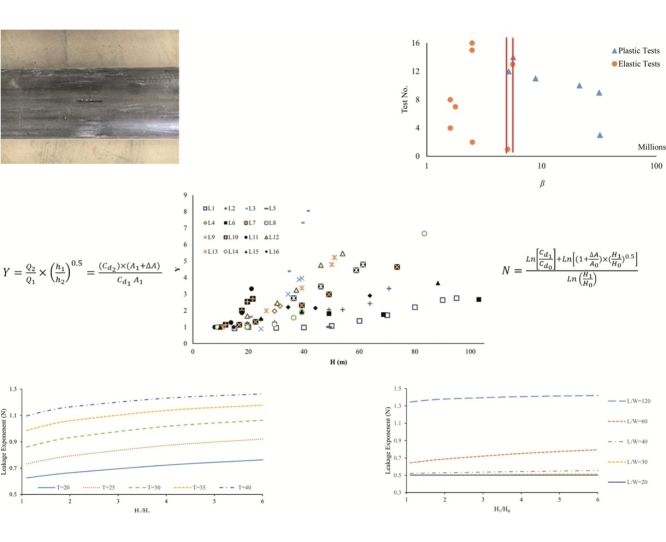

**Specifications Table**

**Table:** 

Subject Area:	*Environmental Science* *Materials Science* *Engineering*
More specific subject area:	*Environmental Sustainable Development and Hydraulic Engineering*
Method name:	*In the present study, longitudinal crack behavior was investigated experimentally by a semi-industrial pilot-scale in HDPE pipes.*
Name and reference of original method:	*Fox, S., R. Collins, and J. Boxall, Experimental Study Exploring the Interaction of Structural and Leakage Dynamics. Journal of Hydraulic Engineering, 2016: p.* 04016080. *Ferrante, M., et al., Experimental evidence of hysteresis in the head-discharge relationship for a leak in a polyethylene pipe. Journal of Hydraulic Engineering, 2010. 137(7): p. 775*–*780.*
Resource availability:	*The data are available with this article*

## Method details

Water resource management is affected by water consumption and it is important to reduce the water losses in water supply systems. Sustainable development needs to focus more on water losses that lead to more energy and water resource consumption. The leakage rate is different in water distribution networks (WDNs) and the leakage varies between 3% (in developed countries) and 50% (in undeveloped countries) of WDNs inflow [1]. The high rate of leakage could disrupt WDNs performance. Recognition of leakage mechanism could help to select an optimum strategy to deal with [2]. High-density polyethylene (HDPE) pipes are extensively applied in WDNs because of ease of installation, inexpensive material, easy transportation and rapid repair. The leakage rate is more sensitive to pressure in plastic pipes [3,4] so that understanding the leakage mechanism in HDPE pipes is essential.

Most leakage in HDPE pipes generally observes in longitudinal slits due to the reduced pipe resistance and increased pressure, however, other leaks such as circumferential slits and orifices can be also occurred through tapping because of external loads or temperature variations [5].

*Torricelli* equation is usually used to estimate the leakage from a slit. This equation which is driven based on the energy balance shows the relationship between leakage and pressure as follows:

(1) $$Q=C_{d}\times A\times \sqrt{2g\times H}$$

Where *Q* is the leakage, *C_d_*: the discharge coefficient, *A*: the leak area, *g*: the gravitational acceleration and *H*: total pressure head. *Torricelli* equation shows the *H* exponent is 0.5 [6,7] but in the field study, it is reported more than 0.5 even as far as 2.79 [8]. *May* introduced a hypothesis to describe the difference [9]. The hypothesis divides the pipe material to rigid and flexible that affect the leak area discharge paths. *FAVAD* theory justifies the difference in leak area (*A*). Plastic pipes classified in flexible categories and the deformation of leak opening in these pipes under internal loading due to pressure depends on different parameters such as the diameter, thickness and material of pipes and also the form of leak openings [3]. Also, increasing *Reynolds* number can decrease the discharge coefficient while by wrapping outward slit edges, the discharge coefficient might be increased [10,11]. Therefore, based on the field studies results a general formula was developed by the International Water Association (IWA) as follows:

(2) $$Q=\;c\times H^{N}$$

Where *c* is leakage coefficient and *N* is leakage exponent. According to *FAVAD* theory, leakage exponent in flexible pipes are greater [12] and it is shown that the difference between *Torricelli* equation and the results of field studies are low in low pressures [13]. A general relation was presented for the explanation the leakage exponent and variable leak area; this relation is based on stress concentration that estimates the maximum value of *N* up to 2.5 [14].

A study showed there is a linear relationship between the leak area (*A*) and pressure head (*H*) in pipes using numerical investigation. Therefore, the maximum leakage exponent is 1.5 in elastic deformation [15].

*C_d_* depends on the shape of leak opening and it is assumed to be a fixed value in most of the previous studies. Flow regimes and turbulence also affect *C_d_* [14] and researchers suggest that since the flow regime of the leakage is almost turbulence, the variation of *C_d_* can be neglected [16]. The average value of *C_d_* is 0.8 for a round orifice and 0.6 for a slit or glottis-shaped orifice in pipes [17]. The leak shape and roughness of its edge could extremely affect the discharge coefficient [18]. The previous research show that some parameters such as thickness, diameter and material of pipes, water pressures and form of leak openings are effective on discharge coefficient [6,16,19,20]. There is no agreement on the value of discharge coefficient for leak opening in the pipes and a wide range between 0.4 and 0.8 is proposed for *C_d_*.

The surrounding environment of the leaks including water, air and soil have also effect on the value and the variations of discharge coefficient [19,21]. The soil around the buried pipe might be removed due to the outlet jet of the leak and the leak opening can be in free flow or submerged condition [3,22].

Despite lots of studies on the leakage in pipes, few studies have been conducted on the plastic pipes especially HDPE. The leakage exponent for the longitudinal slits covers a wide range (0.48≤ *N* ≤ 1.97) and pipe material has a significant impact on the amount of *N* [6,[23], [24], [25]]. The leakage exponent of longitudinal slits in plastic pipes is higher than other material [[26], [27], [28]]. The leakage exponent for longitudinal slits in the HDPE pipe with the diameter of 110 mm has been obtained 0.6 and discharge coefficient was estimated as 0.47 in an experimental study [29]. Leakage mechanism has been studied for small diameter HDPE pipes. The study presents the significant effect of slit length on leakage and IWA relation could predict the leakage rate [7].

Longitudinal slits in Medium-Density Polyethylene (MDPE) pipes were studied with an experiment setup on 63 mm diameter specimens with a 2 bar constant pressure. The pipes showed a viscoelastic behavior and the discharge coefficient was calculated (0.4 ≤ *C_d_* ≤ 0.66) [20]. The results were used to adjust the numerical model and it is presented that the pressure-leakage relation should be modified [30].

Although several experimental [3,20,22], numerical [11,31] and field [6,32] research have been done, the leakage behavior in the HDPE pipes is still arguable.

The difference in the raw materials used for HDPE pipes production, cause to use the Melt Flow Rate (MFR). Higher MFR shows lower tensile strength, softening temperature and elasticity of HDPE pipes. The elasticity of HDPE pipes is sensitive to temperature. Increasing in temperature reduces the elasticity and yield stress in HDPE pipes [33,34].

The leakage and pressure relationship in longitudinal slits is experimentally investigated in HDPE pipes in the present study. The sub-objectives of this research are multifold and include:

• Investigation of discharge coefficient for longitudinal slits in HDPE pipes.
• Examine the temperature effect on the leakage mechanism.
• Define the index of the elastic and plastic behaviors of longitudinal slits in the HDPE pipes.

## Experimental setup

**Fig. 1:**
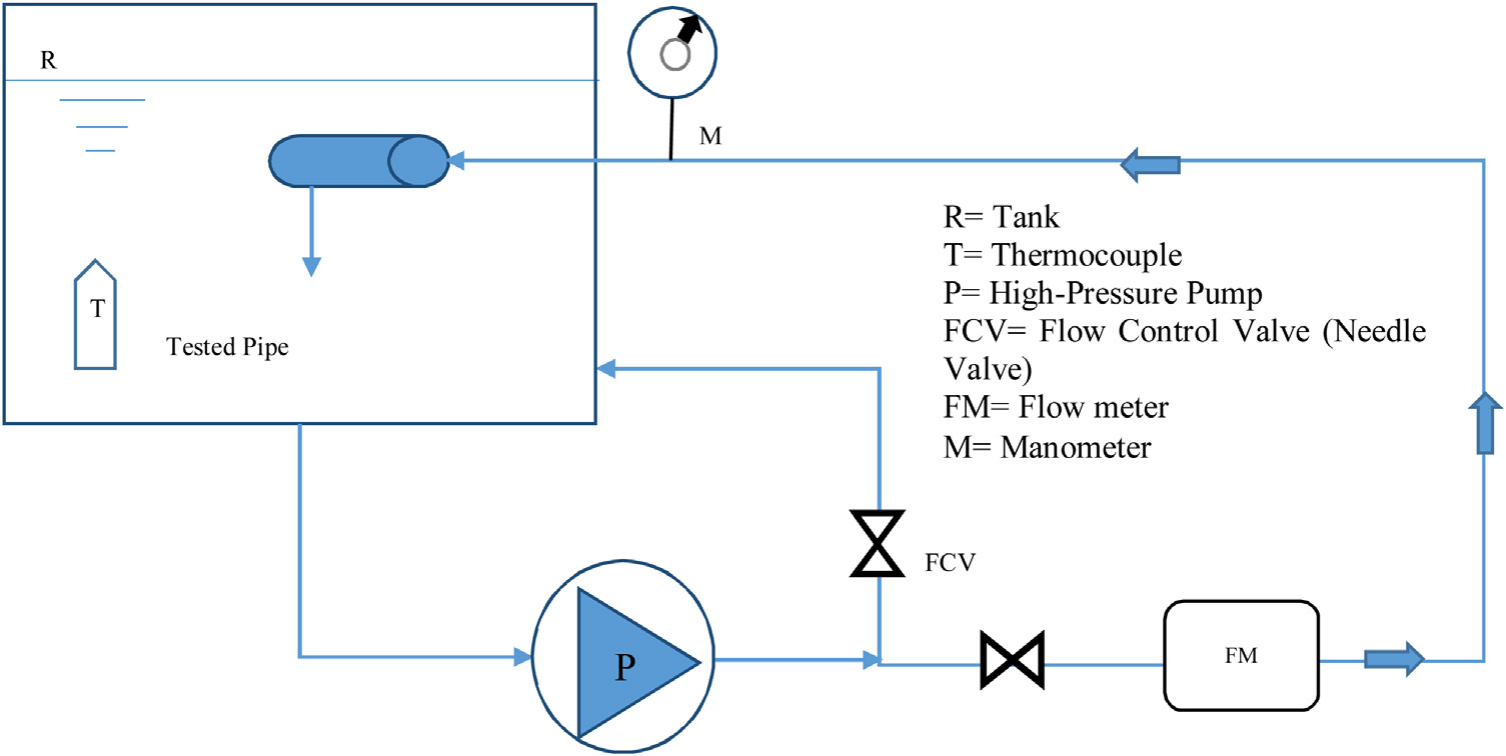
Schematic view of the experimental pilot.

The experimental setup and its details are shown in Fig. 1. The test pipes were located in a tank, in which the leakage flow could be submerged or free condition. Water was directed into the pipe and discharged out by the artificial leak opening created on the HDPE pipe surface. Water pressure was provided by a high-pressure pump and the amount of pressure applied to the test section was set by regulating valves. A pressure gauge with a precision of ±1 (m H_2_O) connected to the inlet cap.

As shown in Fig. 1, water flows in a closed-cycle and the needle valves are used to control the discharge flow and pressure. The pressure was gradually increased in all the experiments. During the experiments, it was observed that the water temperature gradually increased due to pump operation and long duration of each test. The instantaneous water temperature was recorded for the entire duration of the test.

The length of each specimen was 1.20 m and the leak opening was created artificially in the middle of each pipe. Experiments carried out on 16 specimens for the longitudinal slit. The specifications of the test sections are summarized in Table 1.

**Table 1:** Details of the experiments conducted on the longitudinal slit.

Test *No.*	Pressure Head (*m*)	Nominal Pipe Diameter (*mm*)	Pipe Thickness (*mm*)	Slit Length (*mm*)	Slit Width (*mm*)	Temperature (*°C*)	MFR	Pipe Material	Surrounding Environment
L1	15–95	110	7.0	30	1.4	32–40	0.21	PE100	Water
L2	10–72	110	8.3	41	1.2	24–38	0.21	PE100	Water
L3	10–42	110	3.2	29	1	27–35	0.21	PE100	Water
L4	10–32	110	8.1	58	1.2	32–36	0.21	PE100	Water
L5	50	110	8.3	41	1.2	21–36	0.21	PE100	Water
L6	10–105	160	21.1	41	1	31–39	0.46	PE80	Water
L7	10–75	160	13.0	35	1	23–40	0.44	PE80	Water
L8	10–63	160	11.3	40	1	27–40	0.24	PE100	Water
L9	15–40	160	5.2	39	1	26–38	0.45	PE80	Water
L10	10–22	63	3.2	46	1	28–38	0.41	PE80	Water
L11	8–22	63	3.9	48	1	32–37	0.34	PE100	Water
L12	10–55	63	6.1	41	1	30–30	0.45	PE80	Water
L13	11–52	63	5.0	41	1	29–39	0.26	PE100	Water
L14	9–85	250	11.0	29	1	23–32	0.46	PE80	Water
L15	10–90	250	15.5	35	1	32–39	0.46	PE100	Water
L16	15–65	110	8.3	41	1.2	32–40	0.21	PE100	Air

**Fig. 2:**
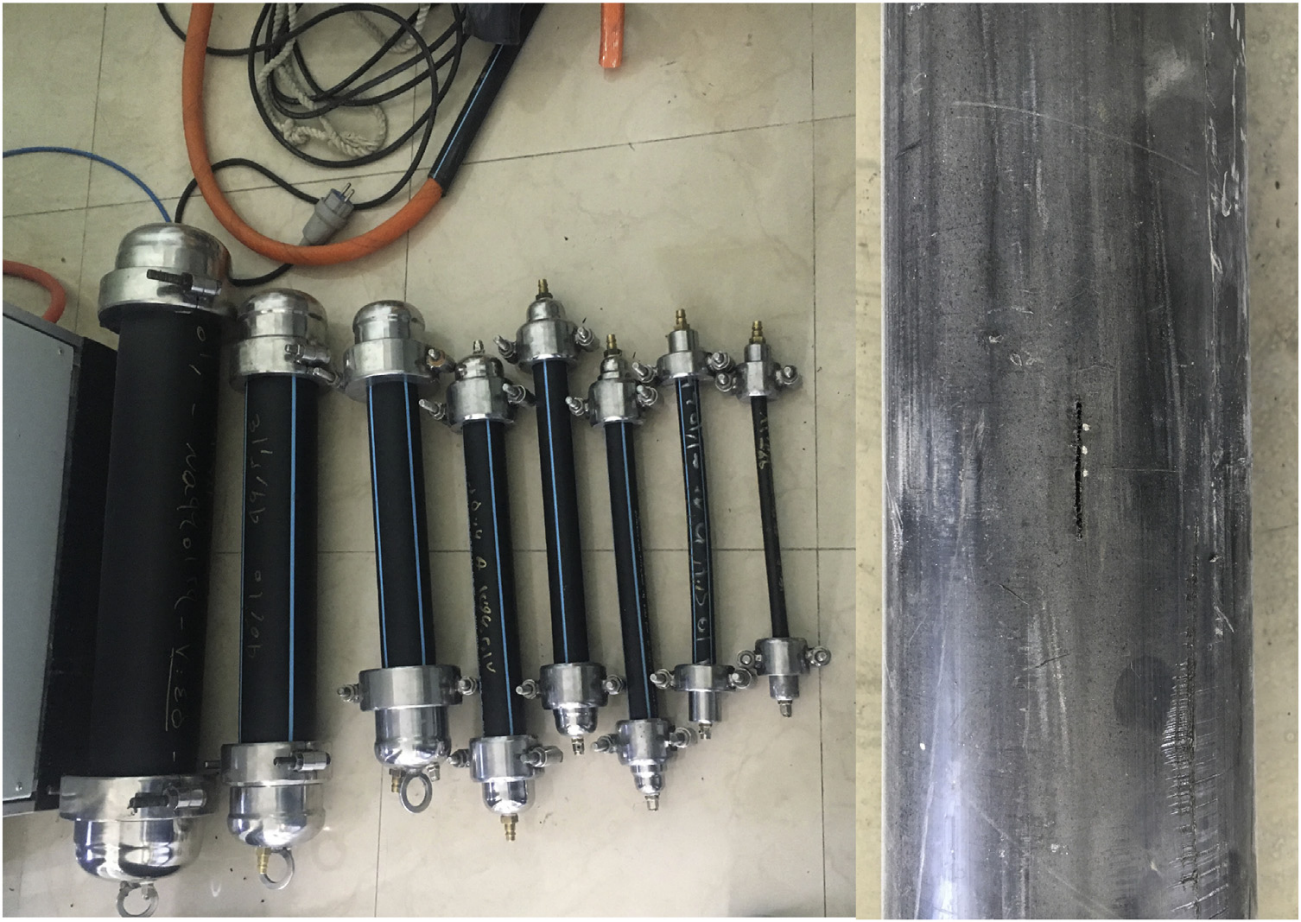
Prepared test section (longitudinal slit).

An electromagnetic meter was applied with ±1% accuracy to measure the leakage flow. The length of leak opening and thickness of pipe are measured by a digital caliper and the width or diameter of slits are measured by a gap-gauge with ±10^−4^ meter precision. Fig. 2a and b shows the prepared specimens.

After applying pressure, enough time was provided so that the flow, the pressure became constant. Then the pressure and flow values were recorded. All experiments were carried out on new pipes to minimize residual stress or deformation.

## Results

Based on Eq. (1), with increasing the pressure head, the leakage discharge increases in the longitudinal slit due to change in water velocity $(\sqrt{2gH}$ (, leak opening area (*A*) or discharge coefficient (*C_d_*). To evaluate simultaneous variations for *A* and *C_d_* with increasing the *H*, the amount of variation in leakage flow is calculated as Eq. (1).

(3) $$Q_{1}=\;C_{d_{1}}\;A_{1}\;\sqrt{2gh_{1}}$$

(4) $$Q_{2}=C_{d_{2}}\;A_{2}\;\sqrt{2gh_{2}}$$

Suppose index "*1*" and "*2*" stand for two different conditions while *H_2_* ≥ *H_1_*; and assuming the leakage area expansion, by dividing the Eq. (3) by 4 and using Eq. (2), *Y* is defined as follows:

(5) $$Y=\frac{Q_{2}}{Q_{1}}\times {\left(\frac{h_{1}}{h_{2}}\right)}^{0.5}=\frac{{(C}_{d_{2}})\times (A_{1}+\Delta A)\;}{C_{d_{1}}\;A_{1}}$$

**Fig. 3:**
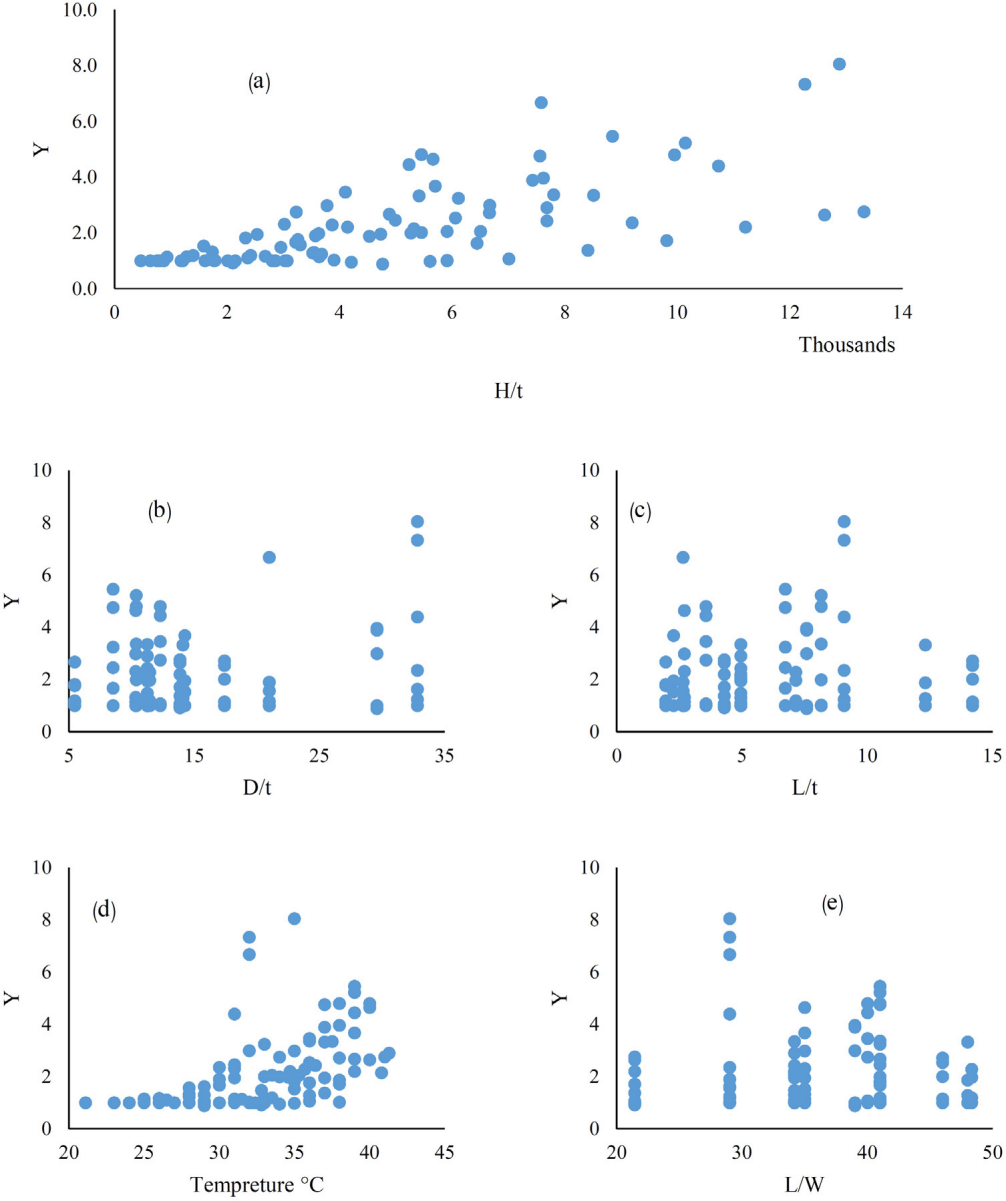
Variations of *Y* in relation to a) pressure to pipe thickness ratio b) diameter to thickness of pipe ratio c) length of slit to pipe thickness ratio d) different temperatures e) length to width of slit ratio.

In Fig. 3 variations of *Y* versus different parameters are shown for all longitudinal slit tests.

Initial discharge coefficient was calculated by Eq. (1) for each test section in which the pressure was minimum. Considering the low pressure in the first test (*H*≤ 10 m), it was assumed that the cross-section of the leak area was constant.

As it is observed in Fig. 3, each specimen has a different behavior against pressure and the rates of variation are also different. As it can be seen the values of *Y* for tests are more than 1 which shows the increase in *A* or *C_d_* by *H*. In some cases, *Y* did not follow the linear behavior as reported by [15]. Data distribution indicates that, in addition to pressure, the leakage flow also depends on other parameters.

### Elastic and plastic mechanism

The results of the present measurements showed that, in none of the current tests, variation in the length of the slits did not occur. To study the elastic deformation of the slits, in each experiment the slit width was measured for 12 h after the unloading. It was observed while for some specimens, the slit width was reversed to its initial width (elastic behavior) for other test sections, the slit had a plastic behavior. The effect of different parameters on the behavior of the longitudinal slit was studied by a detailed dimensional analysis, and the below criteria (*β*) is proposed to distinguished elastic and plastic phases of the longitudinal slits:

(6) $$\beta =\frac{H\times D^{1.5}\times L}{e^{3.5}}\times MFR$$

**Fig. 4:**
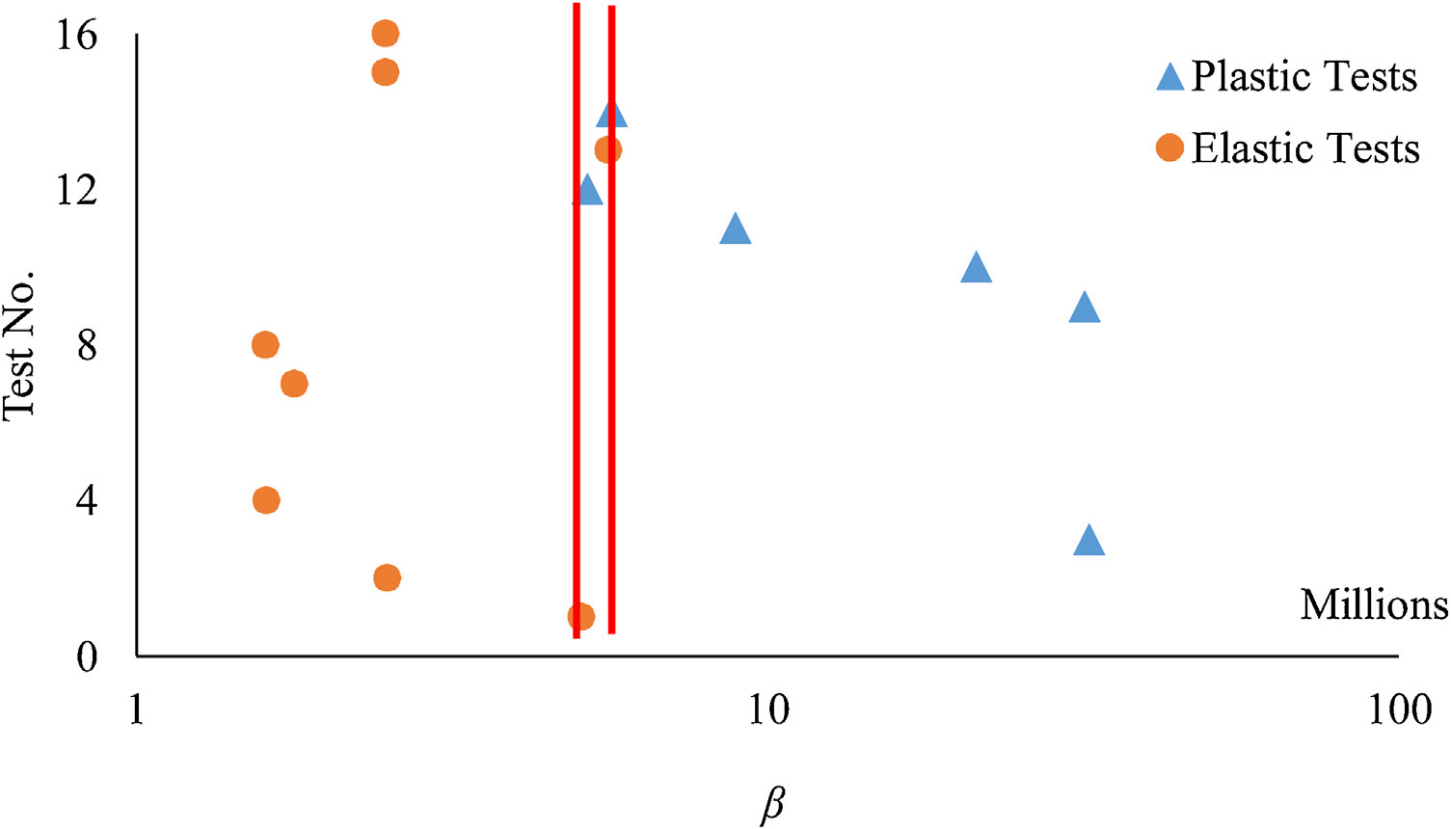
Test section in terms of elastic and plastic behavior of the leakage opening.

Where: *D* is the pipe internal diameter, *e* is the thickness of pipes and *L* is length of longitudinal slits. Fig. 4 divides the elastic and plastic phase of longitudinal slits behavior using the newly defined criteria (*β*).

According to the results, the following criteria are recommended to distinguish elastic and plastic behavior of longitudinal slits in HDPE pipes:

$$\beta <50\times 10^{5}\;\to \;Elastic\;Zone$$

(7) $$50\times 10^{5}<\beta <57\times 10^{5}\to \;Transient\;Zone$$

$$\beta >57\times 10^{5}\to \;Plastic\;Zone$$

Due to the unknown behaviors of leak opening in the plastic phase, hereafter only the elastic data are used. It should be noted that according to the definition of *β*, the status of leakage in HDPE pipes which are normally used in water distribution networks, are in the elastic phase.

### Discharge coefficient

The discharge coefficient for rectangular longitudinal slits in the walls of a thin-walled tank proposed as [35]:

(8) $$C_{d}≅0.59+\frac{8.9}{\sqrt{Re}}\;Re>10^{4}$$

In addition to *Re*, the pipe thickness and hydraulic diameter of slits could affect the discharge coefficient [20].

Analyzing the present experiments showed that in addition to *Re*, the pipe thickness, hydraulic diameter of the slit, temperature also affects the discharge coefficient of the longitudinal slit in HDPE pipes. The edge curvature of the longitudinal slits caused by the increased temperature, pressure and flow in HDPE pipes could increase the coefficient.

Considering the above-mentioned effective parameters, one can write:

(9) $$C_{d_{0}}=\;\varphi \left(Re,\frac{e}{W},t\right)$$

Where: *t* is the temperature and *W* is the slit width.

In the current experiments which *Re* ≥ 1.8 × 10^5^; it was observed that *Re* has an insignificant impact on *C_d_*. The result of the dimensional analysis showed that discharge coefficient for submerged flow can be stated as follows:

(10) $$C_{d}=0.4+0.021\times ({\frac{e}{w})}^{0.69}\times {\left(\frac{t}{20}\right)}^{0.5}\;20\text{℃}\leq t\leq 40\text{℃}\;$$

**Fig. 5:**
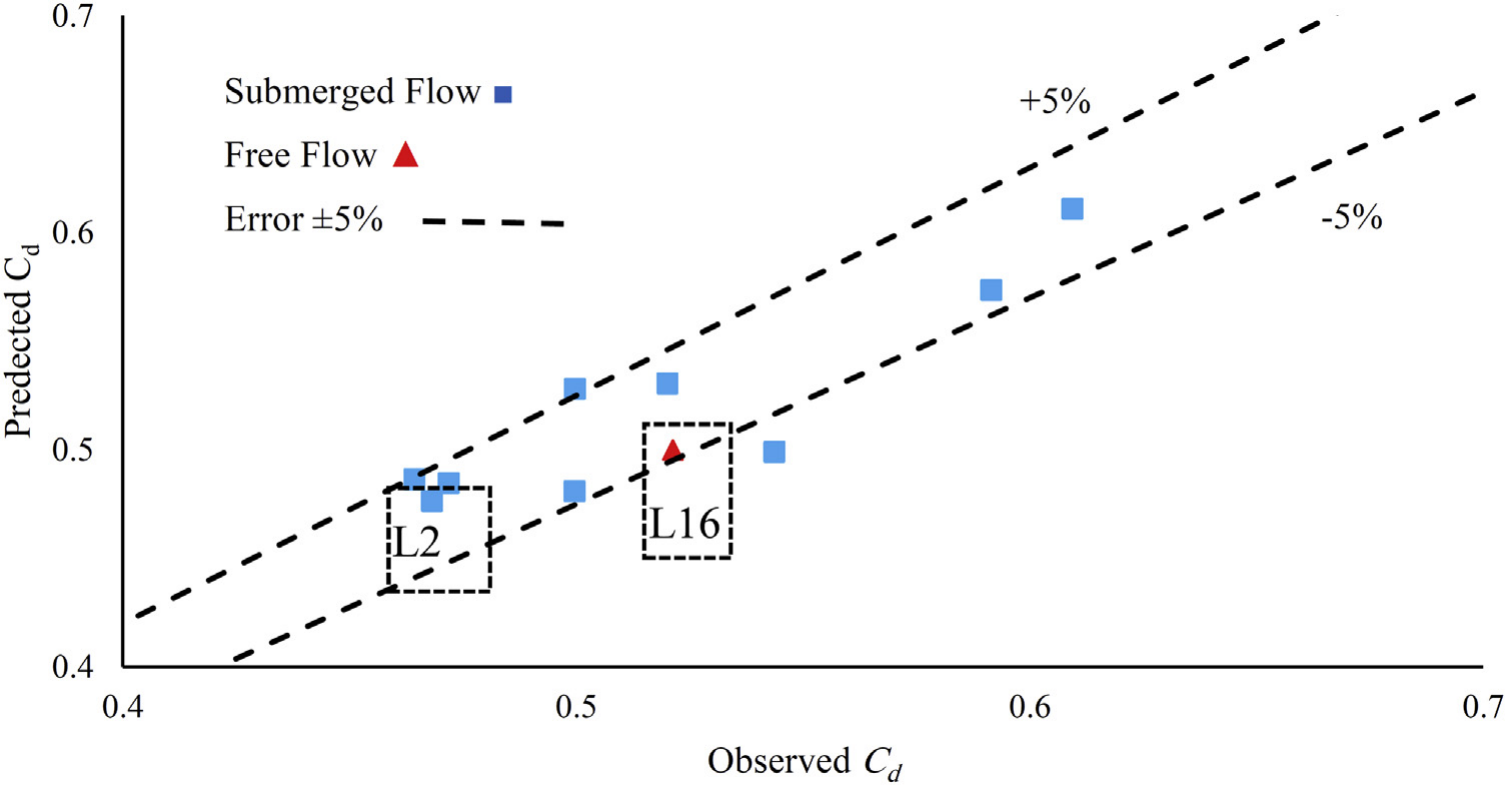
A comparison between the predicted (Eq. (15)) and the observed values.

Where *t* is the temperature in Celsius, which divided by 20 °C in Eq. (10) as a reference temperature. The correlation coefficient for this equation is (*R^2^* = 0.74) and the average error is 4.3%. Fig. 5 compares Eq. (15) with the observed data.

The tests *L16* and *L2* have approximately the same specifications, but they were in different surrounding environment. As it shows in Fig. 5, the discharge coefficient in free flow is higher than the submerged.

### Leakage discharge

By replacing Eq. (10) in Eq. (1), and considering the deformation, the leakage discharge yields:

(11) $$Q=(0.4+0.021\times ({\frac{e}{w})}^{0.69}\times {\left(\frac{t}{20}\right)}^{0.5})\times (A_{0}+\Delta A)\;\sqrt{2gH}$$

Therefore the longitudinal slit deformation (ΔA) is:

(12) $$\Delta A=\frac{Q}{(0.4+0.021\times ({\frac{e}{w})}^{0.69}\times {\left(\frac{t}{20}\right)}^{0.5})\times \sqrt{2gH}}-A_{0}$$

Leakage discharge and other parameters of the Eq. (12) were measured for 64 test so that the predicted value of Δ*A* can be obtained for each test by Eq. (12).

Variations of leak area is depend on various parameters such as pressure, pipe material, slit geometry and temperature. By calculating Δ*A* from Eq. (12), it is possible to develop a new relation between Δ*A* and its effective parameters. By this method after an extensive dimensional analysis of experimental data, the below nonlinear equation is proposed to estimate (Δ*A*):

(13) $$\Delta A=\left[\frac{3.72\times 10^{-13}\times L^{3.27}\times D^{0.85}\times {\left(\frac{t}{20}\right)}^{3.36}\times {MFR}^{0.3}}{e^{1.42}\times W^{1.7}}\times H\right]$$

The correlation coefficient and the average error of Equation 18 with the experimental data are 0.92 and 17.8% respectively. By replacing Eq. (13) into Eq. (11) the general equation for estimation of leakage discharge is proposed as:

(14) $$Q=\left[0.4+0.021\times ({\frac{e}{w})}^{0.69}\times {\left(\frac{t}{20}\right)}^{0.5}\right]\times \left[A_{0}+\frac{3.72\times 10^{-13}\times L^{3.27}\times D^{0.85}\times {\left(\frac{t}{20}\right)}^{3.36}\times {MFR}^{0.3}}{e^{1.42}\times W^{1.7}}\times H\right]\times \sqrt{2gH}$$

**Fig. 6:**
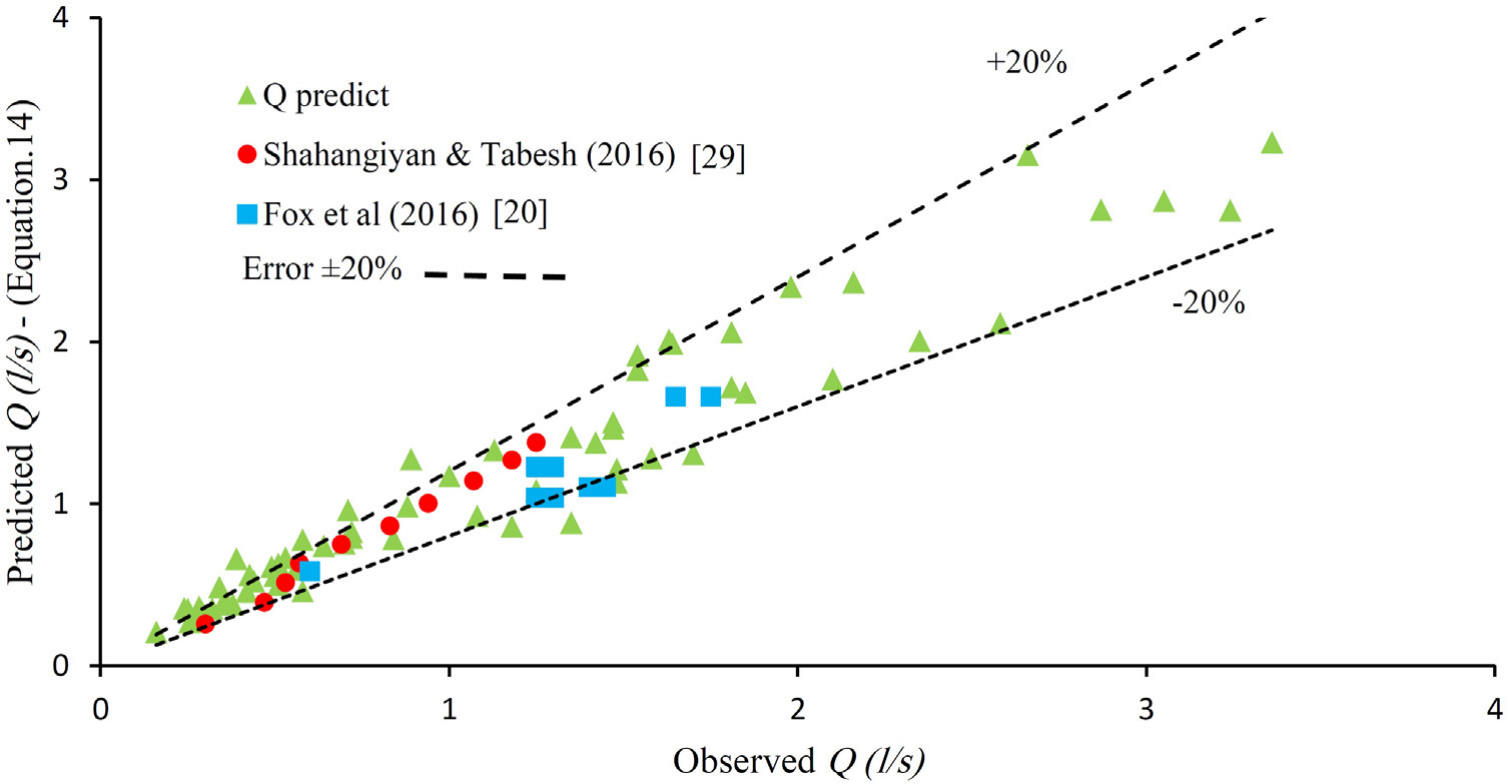
Comparing the predicted leakage discharge (Eq. (19)) with the observed data.

In Fig. 6 the results of Eq. (14) are compared with the present study data. All data in Fig. 6 are within the ±20% error line. In this figure, the experimental results of previous studies [20,29] are also shown for comparison showing the predictability of the proposed equation.

Eq. (14) shows that leak opening area expansion in the longitudinal slit of the HDPE pipe has a linear relation to the pressure. It is also reported for leakage in Unplasticized Polyvinyl Chloride (uPVC) pipe [11]. The length of slits has the most impact on the leakage rate as Eq. (14) suggested. The previous studies confirm the present result [7,11]. Also, the increasing temperature could greatly increase leakage. Considering the frequency of longitudinal slits fault in HDPE pipes, using these pipes could increase the leakage level in warm regions. Moreover, it can be concluded from Eq. (14) that the leakage discharge rate will be lower in pipes with lower MFR.

### Leakage exponent (N)

It is very important to assess the effect of pressure reduction on the leakage rate of WDNs and choose a decision upon. Based on previous studies a wide range for the leakage exponent in WDNs (0.47 ≤ *N* ≤ 2.5) have been suggested [6,23,24]. In the present study, the leakage exponent for elastic phase data was within the range of (0.44 ≤ *N* ≤ 1.44) and it was observed up to 1.95 for the plastic phase.

Moreover, based on the Eq. (14) for the elastic behavior, if the pressure increases, the leakage exponent will tend to 1.5. Writing Eq. (4) for two "*0*" and "*1*" cases and dividing *Q_1_* by *Q_0_* yields:

(15) $$\frac{Q_{1}}{Q_{0}}=\left(\frac{C_{d_{1}}}{C_{d_{0}}}\right)(1+\frac{\Delta A}{A_{0}})\times {\left(\frac{H_{1}}{H_{0}}\right)}^{0.5}$$

Also, writing Eq. (2) for two "*0*" and "*1*" cases and dividing *Q_1_* by *Q_0_* as:

(16) $$\frac{Q_{1}}{Q_{0}}={\left(\frac{H_{1}}{H_{0}}\right)}^{N}$$

The leakage exponent is derived by using the Eqs. (15) and (16) as:

(17) $$N=\frac{Ln\left[\frac{C_{d_{1}}}{C_{d_{0}}}\right]+Ln\left[(1+\frac{\Delta A}{A_{0}})\times {\left(\frac{H_{1}}{H_{0}}\right)}^{0.5}\right]}{Ln\;\left(\frac{H_{1}}{H_{0}}\right)}\;$$

Δ*A* and *C_d_* can be calculated by Eqs. (13) and (10) respectively.

**Fig. 7:**
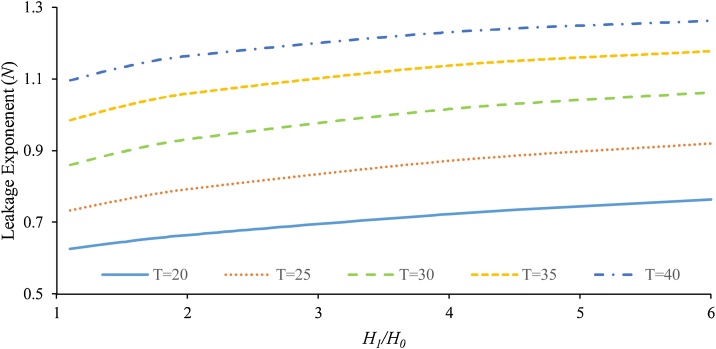
The effect of temperature on leakage exponent for (slit 1 × 60 mm).

**Fig. 8:**
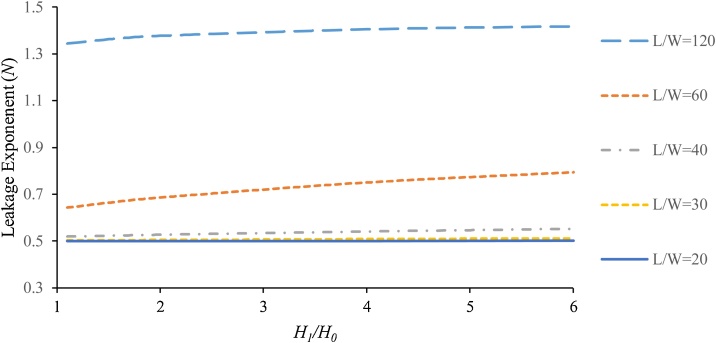
The effect of the slit geometry on leakage exponent at (*t* = 20 °C).

In the field studies, *N* value usually varies across WDNs since the effective parameters such as length and width of the slits are not the same. By using Eq. (17), Fig. 7, Fig. 8 are plotted to show the effects of temperature and *L/W* ratio on the leakage exponent (where *D* = 110 mm, *e* = 8 mm, MFR = 0.2 and the leak opening area = 60 mm^2^).

## Conclusions

In the present research, the leakage rate in the longitudinal slit in the HDPE pipes was experimentally investigated by semi-industrial setup. The results show that seven parameters affecting the leakage rate including diameter, thickness and MFR of the HDPE pipes, forms and dimensions of leak opening, surrounding environment and water temperature. Based on the study, the following results are obtained:

• A new equation was presented to estimate the discharge coefficient for longitudinal slits in HDPE pipes. It was observed that the discharge coefficient in free flow was more than the submerged flow condition. The predicted *C_d_* is less than the value that previous research presented.
• A new criteria have been introduced to evaluate and distinguish between elastic and plastic mechanism of the longitudinal slit in HDPE pipes.
• To estimate the leak area Δ*A* in the elastic phase for the longitudinal slit in HDPE pipes a new experimental equation has been presented as a function of different parameters including temperature and MFR.
• The results show that *N* ≤ 1.5 for the elastic and *N* ≤ 2 for the plastic phase.
